# Periventricular gradient of T_1_ tissue alterations in multiple sclerosis

**DOI:** 10.1016/j.nicl.2022.103009

**Published:** 2022-04-16

**Authors:** Manuela Vaneckova, Gian Franco Piredda, Michaela Andelova, Jan Krasensky, Tomas Uher, Barbora Srpova, Eva Kubala Havrdova, Karolina Vodehnalova, Dana Horakova, Tom Hilbert, Bénédicte Maréchal, Mário João Fartaria, Veronica Ravano, Tobias Kober

**Affiliations:** a Department of Radiology, First Faculty of Medicine, Charles University and General University Hospital, Prague, Czech Republic; b Advanced Clinical Imaging Technology, Siemens Healthcare AG, Lausanne, Switzerland; c Department of Radiology, Lausanne University Hospital and University of Lausanne, Switzerland; d Signal Processing Laboratory (LTS 5), École Polytechnique Fédérale de Lausanne (EPFL), Lausanne, Switzerland; e Department of Neurology and Center of Clinical Neuroscience, First Faculty of Medicine, Charles University and General University Hospital, Prague, Czech Republic

**Keywords:** CS, compressed sensing, DTI, diffusion tensor imaging, EDSS, expanded disability status scale, FLAIR, fluid attenuated inversion recovery, GM, grey matter, GRAPPA, generalized autocalibrating partially parallel acquisitions, HC, healthy controls, MPRAGE, magnetization prepared rapid acquisition gradient echoes, MP2RAGE, magnetization-prepared 2 rapid acquisition gradient echo, MTR, magnetisation transfer ratio, NAGM, normal-appearing grey matter, NAWM, normal-appearing white matter, PPMS, primary progressive multiple sclerosis, SPMS, secondary progressive multiple sclerosis, SST, study specific template, UNI, uniform image, WM, white matter, Multiple sclerosis, Gradient of tissue damage, T_1_-relaxometry, MP2RAGE, Atlas-based assessment

## Abstract

**Objective:**

Pathology in multiple sclerosis is not homogenously distributed. Recently, it has been shown that structures adjacent to CSF are more severely affected. A gradient of brain tissue involvement was shown with more severe pathology in periventricular areas and in proximity to brain surfaces such as the subarachnoid spaces and ependyma, and hence termed the “surface–in” gradient. Here, we study whether (i) the surface-in gradient of periventricular tissue alteration measured by T_1_ relaxometry is already present in early multiple sclerosis patients, (ii) how it differs between early and progressive multiple sclerosis patients, and (iii) whether the gradient-derived metrics in normal-appearing white matter and lesions correlate better with physical disability than conventional MRI-based metrics.

**Methods:**

Forty-seven patients with early multiple sclerosis, 52 with progressive multiple sclerosis, and 92 healthy controls were included in the study. Isotropic 3D T_1_ relaxometry maps were obtained using the Magnetization-Prepared 2 Rapid Acquisition Gradient Echoes sequence at 3 T. After spatially normalizing the T_1_ maps into a study-specific common space, T_1_ inter-subject variability within the healthy cohort was modelled voxel-wise, yielding a normative T_1_ atlas. Individual comparisons of each multiple sclerosis patient against the atlas were performed by computing z-scores. Equidistant bands of voxels were defined around the ventricles in the supratentorial white matter; the z-scores in these bands were analysed and compared between the early and progressive multiple sclerosis cohorts. Correlations between both conventional and z-score-gradient-derived MRI metrics and the Expanded Disability Status Scale were assessed.

**Results:**

Patients with early and progressive multiple sclerosis demonstrated a periventricular gradient of T_1_ relaxation time z-scores. In progressive multiple sclerosis, z-score-derived metrics reflecting the gradient of tissue abnormality in normal-appearing white matter were more strongly correlated with disability (maximal rho = 0.374) than the conventional lesion volume and count (maximal rho = 0.189 and 0.21 respectively). In early multiple sclerosis, the gradient of normal-appearing white matter volume with z-scores > 2 at baseline correlated with clinical disability assessed at two years follow-up.

**Conclusion:**

Our results suggest that the surface-in white matter gradient of tissue alteration is detectable with T1 relaxometry and is already present at clinical disease onset. The periventricular gradients correlate with clinical disability. The periventricular gradient in normal-appearing white matter may thus qualify as a promising biomarker for monitoring of disease activity from an early stage in all phenotypes of multiple sclerosis.

## Highlights

• T_1_ relaxation times alterations were assessed based on a study-specific atlas.
• T_1_ alterations depend on distance from lateral ventricles (“periventricular gradient”).
• Gradient parameters correlate better with disability compared to conventional MRI.

## Graphical abstract

**Figure:**
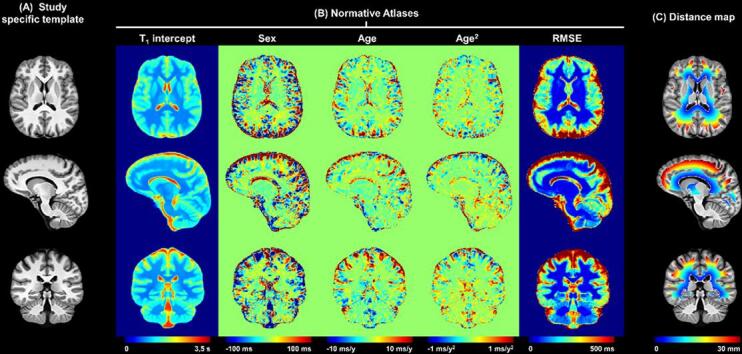

## 1 Introduction

Multiple sclerosis is the most common chronic neurodegenerative and neuroinflammatory disease of the CNS in young adults (Compston and Coles, 2008), and is responsible for irreversible CNS injury and physical disability due to focal demyelination and diffuse structural damage of normal-appearing white (NAWM) and grey matter (NAGM) (Lassmann, 2019). The accumulation of focal demyelinating lesions and damage to normal-appearing tissue in the multiple sclerosis brain does not affect all regions equally (Haider et al., 2016). Lesions preferentially localize in periventricular and juxtacortical white matter, but also in the brainstem cerebellum, optic nerve, and spinal cord (Popescu and Lucchinetti, 2012). This pattern of distribution is in line with recent results from histopathological analyses and advanced imaging studies that have revealed a relationship between the microstructural tissue damage in the cortex, periventricular areas, deep grey matter (GM), and spinal cord with distance from CSF. In particular, the regions adjacent to the inner and outer CSF spaces seem to be more prone to tissue damage as assessed by various quantitative MRI metrics (Liu et al., 2015) (Brown et al., 2017) (Pardini et al., 2019) (De Meo et al., 2021). Several recent quantitative MRI and PET studies have demonstrated the presence of this gradient in the periventricular NAWM (Liu et al., 2015) (Moccia et al., 2020) (Pardini et al., 2016) (Bodini et al., 2020) (Poirion et al., 2021) (Pardini et al., 2021), cortex (Samson et al., 2014), and spinal cord (Combès et al., 2019). The biological mechanism underlying the relationship between distance from CSF and tissue damage is not fully understood yet, and it is remains unclear whether all the different types of pathology gradient share the same pathophysiological mechanism. Currently, the most probable explanation is the association with the CSF-derived proinflammatory cytokines, molecules that stimulate B-lymphocyte activation and the involvement of activated microglia (Magliozzi et al., 2010) (Poirion et al., 2021). In the cortex and spinal cord, the source of these cytokines might be meningeal foci of inflammation composed of B-lymphocytes and dendritic cells (Brown and Sawchenko, 2007).

In terms of MRI techniques, such gradients have been demonstrated using magnetisation transfer imaging (Liu et al., 2015) (Brown et al., 2017) (Moccia et al., 2020), quantitative T2* (Mainero et al., 2015), diffusion tensor imaging (Cacciaguerra et al., 2021), and PET (Poirion et al., 2021). While a novel marker assessing axonal density, the Composite Hindered and Restricted Model of Diffusion (CHARMED), also showed a dependence on distance from the ventricles, it did not differentiate between MS patients and healthy controls (De Santis et al., 2019). To our knowledge, periventricular tissue damage gradients have not been studied by means of quantitative T_1_ mapping. T_1_ relaxation times depend on the integrity of micro and macrostructural tissue components and in MS lesions and NAWM have been found to be prolonged by demyelination, axonal loss, oedema, and decreased iron content, and similarly in deep GM, especially in the thalamus and cortical GM (Mottershead et al., 2003) (Jonkman et al., 2015). T_1_ relaxation times increase with disease progression, and have been shown to correlate with both brain atrophy and physical disability (Vrenken et al., 2006) (Vrenken et al., 2006), and cognitive impairment (Simioni et al., 2014). Up to now, T_1_ mapping has not been included in routine multiple sclerosis MRI protocols, mainly due to technical challenges and only moderate reproducibility. However, the magnetization prepared 2 rapid acquisition gradient echoes (MP2RAGE) sequence has been shown to provide highly reproducible T_1_ values (Marques et al., 2010) (Marques et al., 2013) while maintaining clinically feasible scan times (8 min for a whole-brain scan with 1 mm^3^ isotropic resolution). Even shorter scan times (4.5 min) can be achieved by accelerating the sequence with compressed sensing (Mussard et al., 2020). T_1_ relaxometry can thus be easily integrated into routine clinical MRI protocols. The implementation of T_1_ mapping in clinical practice requires a better understanding of the relation between T_1_ changes and the underpinning tissue structure alterations, and an investigation of how well the association of such T_1_ changes with clinical outcomes compares with conventional MRI metrics.

This study investigates whether the periventricular gradient of tissue alteration found previously with other imaging biomarkers is also reflected by T_1_ relaxometry measurements. More specifically, the aims of our work are fourfold:

(i) To investigate the occurrence of T_1_ relaxation time abnormalities assessed with a recently developed method based on z-scores derived from the voxel-wise comparison of single-subject data to a normative atlas, as an alternative to solely investigating the absolute T_1_ relaxation times.
(ii) To use this novel method to explore in patients with early and progressive multiple sclerosis the presence of a periventricular gradient of tissue damage, characterized by T_1_ relaxation time abnormalities being strongest close to the lateral ventricles and then gradual decreasing with distance from the ventricles.
(iii) To explore whether there is any difference in observed periventricular T_1_-gradients, in either NAWM or focal lesions, between patients with early and progressive multiple sclerosis, and similarly between patients with different levels of physical disability.
(iv) To compare the strength of correlations between physical disability and periventricular T_1_-gradients in NAWM to the correlations of conventional MRI-derived metrics, such as total lesion count and volume.

## 2 Material and methods

### 2.1 Participants

We enrolled 47 patients with a first clinical symptom suggestive of multiple sclerosis (mean age 31.8 ± 8.0 years, 75 % female, disease duration 4.7 ± 5.4 months, median Expanded Disability Status Scale (EDSS) 2.0 [range: 1.0–3.5]) who fulfilled the revised McDonald 2017 criteria (Thompson et al., 2018), and 52 patients with either secondary progressive multiple sclerosis (SPMS; n = 45) or primary progressive multiple sclerosis (PPMS; n = 7) (mean age 49.9 ± 7.2 years, 65 % female, disease duration 19.48 ± 7.8 years).

In addition, we recruited 92 healthy controls (HC) without history of neuropsychiatric diseases or other medical conditions affecting brain health (mean age 37.3 ± 10.6, 63 % female).

All participants signed a written informed consent to participate in a clinical and imaging study approved by the local ethics committee (for patients NV18-04–00168 and for healthy controls NCT03706118). The study was conducted in accordance with the Declaration of Helsinki.

### 2.2 Clinical assessment

All patients underwent a neurological examination and were scored using the EDSS (Kurtzke, 1983). Early multiple sclerosis patients had follow-up over two years, with EDSS assessed at baseline, month 6, month 12, and month 24. We have only used the baseline and month 24 EDSS assessments in the statistical analysis. The EDSS was assessed at least 30 days after the administration of high-dose corticosteroids and outside of any clinical relapse. For patients with progressive MS, the mean interval between MRI and clinical visit with EDSS assessment was 0.93 ± 1.03 months (minimum 0 and maximum 3.8 months).

### 2.3 MRI acquisition

Cohorts of early multiple sclerosis, progressive multiple sclerosis, and HC underwent MRI at 3 T (MAGNETOM Skyra, Siemens Healthcare, Erlangen, Germany) using a standard 20 channel head coil. The protocol included 3D magnetization-prepared 2 rapid acquisition gradient echoes (MP2RAGE) for T_1_ mapping, along with 3D fluid-attenuated inversion recovery (FLAIR-SPACE) and magnetization-prepared acquisition gradient echo (MPRAGE) for brain tissue and lesion segmentation. The sequence parameters are summarized in Supplementary Table 1.

The MRI examination of patients with early multiple sclerosis was that done for diagnostic purposes when multiple sclerosis was suspected. The scanning was done before lumbar puncture and before administration of corticosteroids or immunomodulatory medication.

Demographic, clinical and MRI characteristics of the recruited participants are reported in Table 1. Within the MRI characterization process, a neuroradiologist with 22 years of experience checked that the T2 focal hyperintensities were related to MS; atypical lesions were not found in any of the two cohorts. Visual inspection was also used to check for any severe motion artifacts that might impact image processing, but none were present.

**Table 1:** Demographic and MRI characteristics of the 3 groups.

	Healthy volunteers	Early multiple sclerosis	Progressive multiple sclerosis	Healthy vs. Early MS	Healthy vs. progressive MS	Early MS vs. progressive MS
n	92	47	52			
% Female	63	75	65			
Age (years)	37.3 ± 10.6	31.8 ± 8.0	49.9 ± 7.2	p = 0.24 ^KW^	p < 0.001 ^KW^	p < 0.001 ^KW^
Disease duration*	–	4.7 ± 5.4 **months**	19.48 ± 7.8 **years**	–	–	p < 0.001 ^MW^
EDSS	–	2.0 (1.0 – 3.5)	5.5 (3.5 – 6.5)	–	–	p < 0.001 ^MW^
T2 lesion volume (ml)	–	6.7 ± 8.7	14.1 ± 15.2	–	–	p < 0.001 ^MW^
T2 lesion count	–	22 (1–128)	28 (3–68)	–	–	p < 0.001 ^MW^
Brain volume (%)	80.0 ± 2.2	79.4 ± 2.9	74.9 ± 3.8	p = 1.0 ^KW^	p < 0.001 ^KW^	p < 0.001 ^KW^
Lateral ventricles (%)	1.3 ± 0.4	1.5 ± 0.6	2.2 ± 1.2	p = 0.001 ^KW^	p < 0.001 ^KW^	p = 0.025 ^KW^
White matter (%)	31.7 ± 1.6	31.0 ± 2.4	29.9 ± 2.7	p = 0.308 A	p < 0.001 A	p = 0.049 A
Gray matter (%)	48.3 ± 2.3	48.4 ± 2.2	45.0 ± 2.0	p = 0.9 A	p < 0.001 A	p < 0.001 A

Legend: All volumes are shown as % of total intracranial volume, except for lesion volumes which are in millilitres, and lesion count. All measures are mean ± SD except lesion count (median, range). EDSS = Expanded Disability Status Scale.

*Disease duration is shown in years for patients with progressive multiple sclerosis and in months for patients with early multiple sclerosis.

A = ANOVA; ^KW^ = Kruskal-Wallis test; ^MW^ = Mann Whitney *U* test.

### 2.4 Image analysis

#### 2.4.1 Segmentation of brain tissues on T1 3D MPRAGE

The MorphoBox prototype was used for automated segmentation of brain anatomical structures (Schmitter et al., 2015). The first processing step consisted of an atlas skull stripping followed by an atlas free brain tissue classification (WM, GM, CSF). Global and regional brain structure volumes were then estimated by combining the tissue probability maps obtained in the previous step with anatomical masks derived from a single-subject template-based non-rigid registration.

#### 2.4.2 Lesion and NAWM segmentation

Total volume and count of brain WM lesions were derived from 3D FLAIR and MPRAGE using a fully automated segmentation pipeline (LeMan-PV prototype software, Siemens Healthcare, Erlangen, Germany) (Fartaria et al., 2016) (Fartaria et al., 2019). The resulting lesion mask was dilated using a cubic structuring element of radius 1 and subtracted from the previously computed WM mask using MorphoBox in order to obtain a segmentation mask of the normal-appearing WM (NAWM) for each subject.

#### 2.4.3 Computation of T_1_ z-score maps

**Fig. 1:**
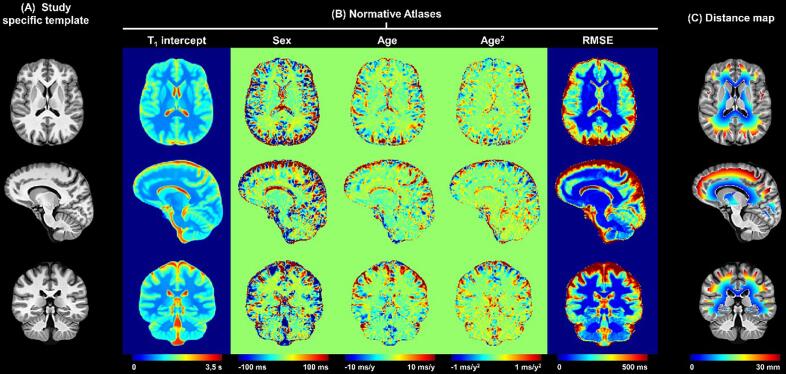
Computation of T_1_ z-score maps and periventricular gradient extraction. Representative slices of (A) the anatomical study-specific template, (B) the normative T_1_ atlas, and (C) the distance to ventricles of the supratentorial WM voxels. The intercept, sex, age, and age^2^ maps in (B) are intended as the β_0_, β_sex_, β_age_ and β_age2_ coefficients of the following equation describing the expected reference T_1_ value in a given brain voxel: $E\left\{T1\right\}=\beta_{0,T1}+\beta_{\mathrm{sex},T1}*sex+\beta_{\mathrm{age},T1}*age+\beta_{age2,T1}*{\mathrm{age}}^{2}$, with sex being a categorical variable and the age expressed in years and centred at the mean age of the healthy control cohort (37 y). The first band was excluded to minimise the partial volume effect.

T_1_ deviations from reference values established in healthy controls were computed in the two patient cohorts. To that end, the healthy controls’ MP2RAGE data (UNI image and T_1_ map) and their respective segmentation masks were spatially normalized into a study-specific template (SST) built from 20 randomly selected healthy controls (Avants et al., 2011). A reference T_1_ $\left(\vec{r}\right)$ atlas of healthy tissue was established by linear, voxel-wise modelling of the T_1_ inter-subject variability, including age and sex as covariates:

$$E\left\{T1\left(\vec{r}\right)\right\}=\beta_{0}\left(\vec{r}\right)+\beta_{\mathrm{sex}}\left(\vec{r}\right)*sex+\beta_{\mathrm{age}}\left(\vec{r}\right)*age+\beta_{{\mathrm{age}}^{2}}\left(\vec{r}\right)*{\mathrm{age}}^{2},$$

with E being the expected value, $\beta_{0}$ being the model intercept, and *sex* a categorical variable equal to 1 if the subject is male or 0 if female (Fig. 1) as detailed in Piredda et al. (2020).

MP2RAGE data of the two multiple sclerosis cohorts were spatially registered onto the SST, and T_1_ deviations from the established normative atlases were assessed using z-scores, evaluated voxel-wise as the following ratio: ($T1\left(\vec{r}\right)-E\left\{T1\left(\vec{r}\right)\right\}$)/RMSE, with $T1\left(\vec{r}\right)$ being the T1 estimated in the patient data, $E\left\{T1\left(\vec{r}\right)\right\}$ the expected T1 from the linear model, and RMSE the root mean squared error of the model (Piredda et al., 2020).

#### 2.4.4 Periventricular gradient extraction

To study the periventricular gradient of tissue abnormalities identified with T_1_ z-scores, one-voxel thick bands were defined around the ventricles in the SST. To that end, ventricle probability masks of the healthy cohort were averaged and then a threshold of p = 50% was applied to obtain the final ventricle mask. Subsequently, the Euclidean distance to the closest voxel in the ventricle mask was assigned to each voxel in the supratentorial WM. The infratentorial regions were excluded from the analysis (due to the small distance between 3rd and 4th ventricle and outer brainstem), consistent with previous work (Liu et al., 2015) (Brown et al., 2017). Distances were then rounded to the nearest integer; following this procedure, 30 periventricular bands were extracted, representing WM layers 1 to 30 mm from the ventricles (Fig. 1). To minimize the partial volume effect, the first band (adjacent to lateral ventricles) was excluded from the analysis. Additionally, the infratentorial parts of the ventricle masks were excluded.

#### 2.4.5 Z-score derived metrics

As this was an explorative study, we assessed several z-score derived metrics computed from individual patient datasets and, for the periventricular bands, separately for the early and progressive multiple sclerosis cohorts:

• Mean values of absolute z-scores > τ in NAWM
• Volume of NAWM voxels with z-score > τ (normalized with regard to the corresponding band volume)
• Mean values of absolute z-scores > τ in lesions
• Volume of voxels in lesions with z-scores > τ,

for τ varying between 0 and 3 (Supplementary Fig. 1).

For all z-score derived metrics, gradients were calculated as a difference (in volume or in mean z-score) between pairs of bands and divided by the distance between the bands. In the results section, we present only the metrics that showed most pronounced differences between early and progressive patients, and which had the highest correlations with EDSS.

### 2.5 Statistical analysis

Statistical analyses were performed with SPSS (version 20., IBM Inc., USA) and R (R Foundation for Statistical Computing, version 3.5.0, Vienna, Austria). Demographic and clinical data are presented as mean ± standard deviation (SD), while EDSS scores and absolute changes are presented as median (range). For inferential statistics, results are reported as mean ± SD, except when specified otherwise. For all tests, the level of statistical significance was set at p < 0.05. To assess the normality of the distributions, we used visual inspection of histograms, Q-Q plots, and the Shapiro–Wilk test. Differences between demographic parameters and global and regional MRI volumes were determined using a Mann-Whitney-*U* test, ANOVA, or a Kruskal-Wallis H-test, followed by Bonferroni or Tukey’s post hoc tests for multiple comparisons. Estimated gradients were compared between the early and progressive multiple sclerosis patients with a two-sided Wilcoxon rank sum test. Spearman’s correlation between all investigated MRI-derived metrics and EDSS were assessed.

### 2.6 Data availability statement

A research dataset that includes tables with absolute T1 values in healthy individuals, absolute T1 values in normal appearing white matter and lesions of patients with early and progressive multiple sclerosis, mean z-scores, and volume of voxels above particular z-score thresholds is available online (Mendeley Data: T1 periventricular gradient - https://data.mendeley.com/datasets/2jg9hmdhhf/1). The normative atlas and the individual T1 maps of patients are available upon request. The request necessitates that the purpose of data re-analysis is in line with the study aims as approved by the ethics review board and consented to by participants. Furthermore, consent to data privacy needs to be assured by signing an appropriate agreement form.

## 3 Results

### 3.1 Periventricular gradient of T_1_ relaxation times in early and progressive multiple sclerosis

**Fig. 2:**
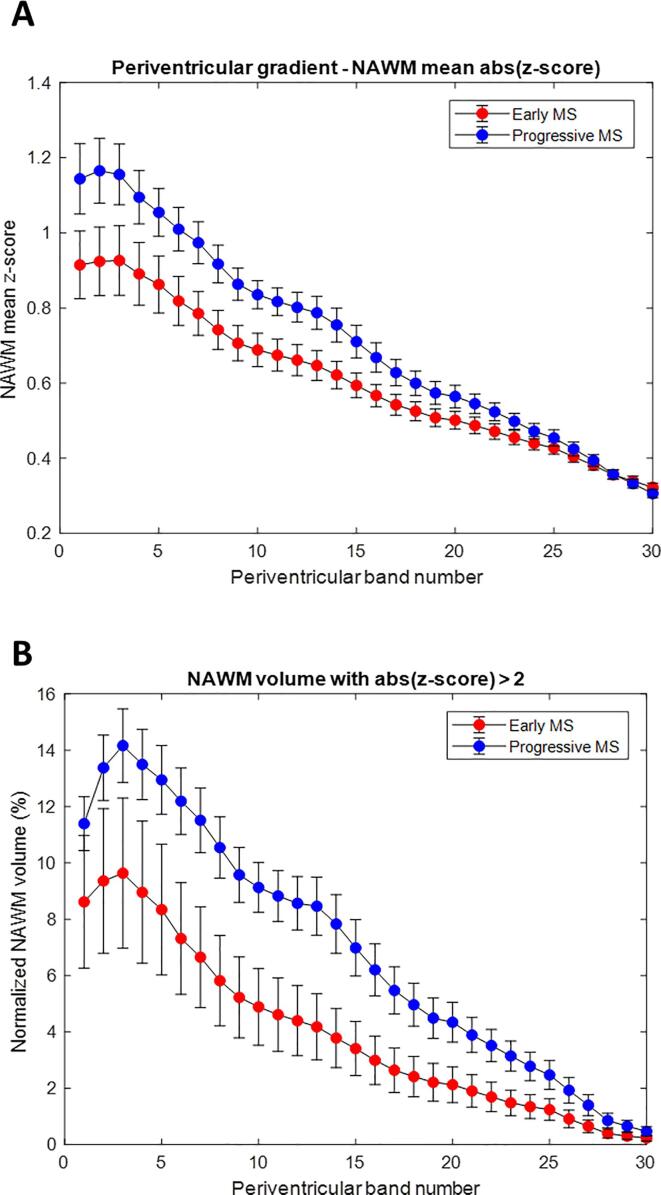
Periventricular gradients in NAWM for mean absolute z-score (exceeding the prediction interval of the T_1_ normative linear model at a 95% level of confidence) and for volume with absolute (z-score) > 2. (A) mean absolute z-scores, (B) volume with absolute (|z-score|) > 2. Error bars indicate two standard errors.

We observed a periventricular gradient of T_1_ abnormalities in both early and progressive MS patients. Fig. 2 shows a comparison of z-score derived metrics in NAWM in early and progressive multiple sclerosis. The most pronounced differences between z-score-derived metrics were observed for the volume of NAWM with z-scores > 2. Similarly, we detected a gradient of mean absolute z-score values with a periventricular maximum and gradual decrease with distance. The absolute z-score values in NAWM were higher in the progressive cohort. Surprisingly, the cohorts did not differ either in absolute z-scores or in z-score-derived per band gradients within lesions. Further, we observed a “gradient” of lesion probability (i.e., lesion volume per band), with higher values closer to the ventricles.

### 3.2 Correlation between physical disability and periventricular gradient

#### 3.2.1 Progressive multiple sclerosis patient cohort

**Fig. 3:**
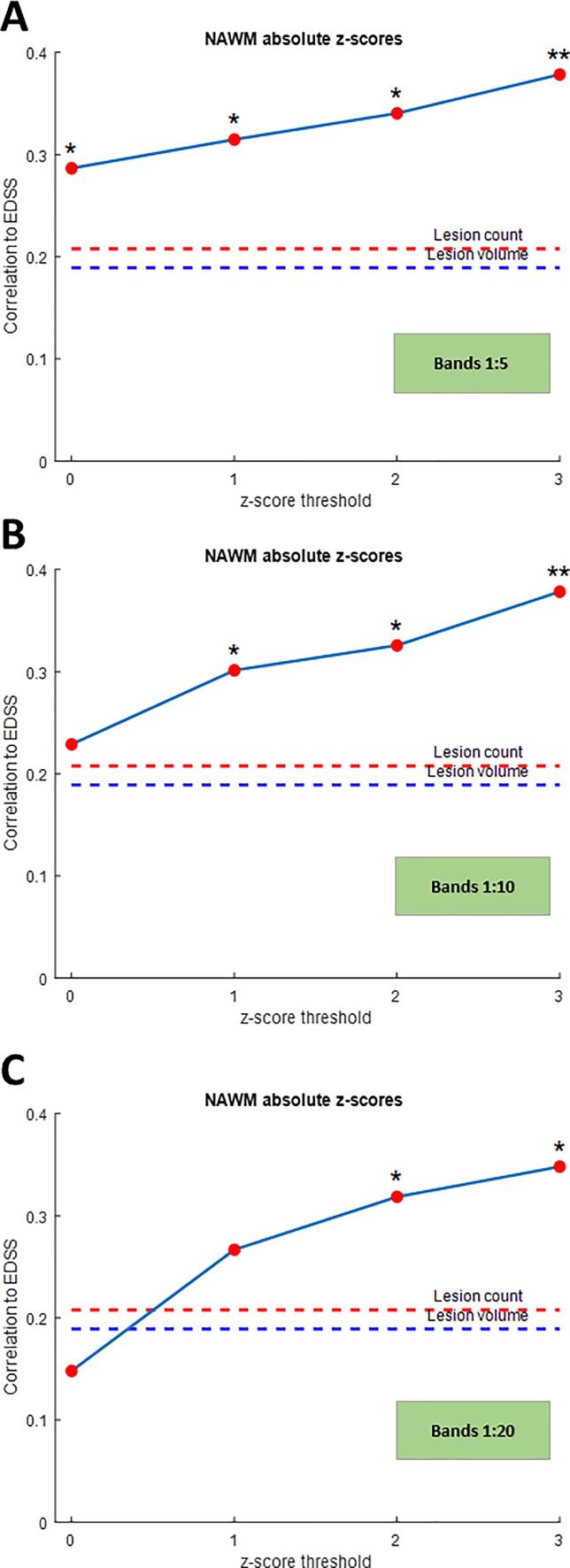
Correlation between gradient of z-scores in NAWM and EDSS (change between bands for z-score thresholds of 0, 1, 2 and 3) in patients with progressive multiple sclerosis: (A) band 1:5; (B) band 1:10 (C) band 1:20. (* indicates statistically significant values).

The conventional MRI derived metrics did not correlate well with EDSS (Spearman’s rho (ρ) for lesion count = 0.208, p = 0.140; ρ for lesion volume = 0.189, p = 0.180.). However, z-score-derived gradient metrics in NAWM showed better correlation with EDSS (Table 2 and Fig. 3). This was particularly true for the mean z-score gradient using an absolute z-score threshold of 2 between bands 1 and 2 (ρ =.374p = 0.006), 1 and 5 (ρ =.341p = 0.014), 1 and 10 (ρ = 0.326, p = 0.018), and 1 and 20 (ρ = 0.319, p = 0.02). The z-score-derived gradient metrics within lesions also correlated with EDSS; the highest correlation was between bands 1 and 20 (ρ = 0.325, p = 0.019) and 1 and 30 (ρ = 0.328, p = 0.018) (Table 3).

**Table 2:** Correlation between gradient of z-scores in NAWM (difference of mean z-scores between the bands divided by distance between bands) and EDSS in patients with progressive multiple sclerosis (* indicates statistically significant values).

	Correlation coefficient for the z-score thresholds				p-values			
Gradient between bands	0	1	2	3	0	1	2	3
1–2	0.325	0.331	0.374	0.390	0.019*	0.016*	0.006*	0.004*
1–5	0.287	0.315	0.341	0.379	0.039*	0.023*	0.014*	0.006*
1–10	0.229	0.301	0.326	0.378	0.103	0.030*	0.018*	0.006*
1–20	0.148	0.267	0.319	0.348	0.294	0.056	0.021*	0.011*
1–30	0.117	0.267	0.306	0.314	0.409	0.056	0.027*	0.023*

EDSS = expanded disability status scale, NAWM = normal appearing white matter.

**Table 3:** Correlation between gradient of z-scores in lesions (difference of mean z-scores between the bands divided by distance between bands) and EDSS in patients with progressive multiple sclerosis (* indicates statistically significant values).

	Correlation coefficient for the z-score thresholds				p-values			
Gradient between bands	0	1	2	3	0	1	2	3
1–2	0.265	0.210	0.194	0.182	0.057	0.135	0.168	0.196
1–5	0.257	0.220	0.201	0.200	0.065	0.117	0.152	0.156
1–10	0.307	0.305	0.310	0.321	0.027*	0.028*	0.025*	0.020*
1–20	0.294	0.310	0.325	0.328	0.034*	0.026*	0.019*	0.018*
1–30	0.303	0.307	0.328	0.315	0.029*	0.027*	0.018*	0.023*

EDSS = expanded disability status scale.

#### 3.2.2 Early multiple sclerosis patient cohort

**Fig. 4:**
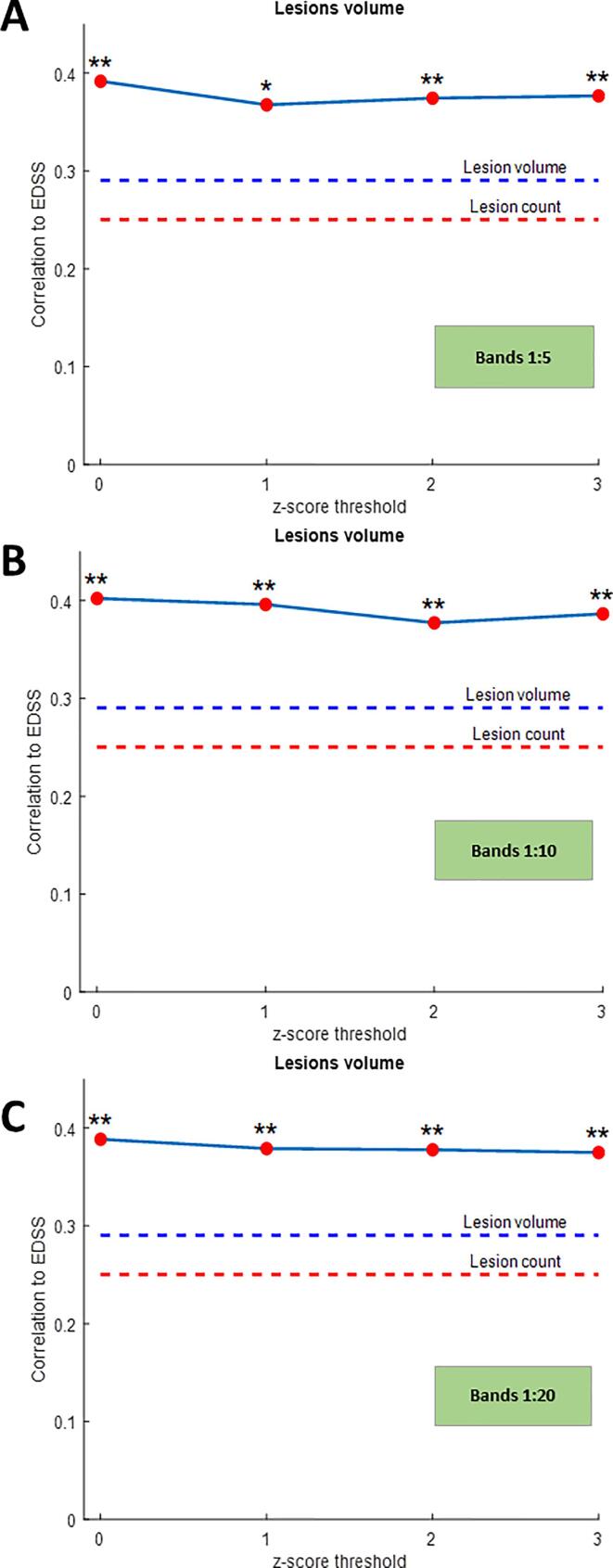
Correlation between gradient of lesion volume exceeding a given z-score threshold at baseline and EDSS assessed 2 years after baseline (change between bands for z-score thresholds of 0, 1, 2 and 3) in patients with early multiple sclerosis: (A) band 1:5; (B) band 1:10; (C) band 1:20. (* indicates statistically significant values, * p < 0.05 ** p < 0.01).

The correlation of EDSS with z-score-derived gradient metrics in NAWM at baseline was higher than with conventional MRI-based metrics, but not statistically significant (Supplementary Fig. 2). We found significant correlation between the gradient of lesion volume exceeding a given z-score threshold at baseline and EDSS assessed 2 years after baseline. The highest correlation was observed for the gradient between bands 1 and 20 (ρ =.335, p = 0.021) at a z-score threshold of 2; the correlations for gradients between bands 1 and 5 and bands 1 and 30 also had a stronger correlation compared with conventional MRI-derived metrics (Spearman’s rho (ρ) for lesion count = 0.160, p = 0.284; ρ for lesion volume = 0.207, p = 0.164), see Fig. 4 and Table 4A. For z-score-derived gradient metrics of the NAWM tissue exhibiting z-score values > 2 at baseline, we also found stronger correlations between EDSS assessed 2 years after baseline and the gradients between bands 1–20, and 1–30 (Table 4B).

**Table 4:** A, B Correlation between gradient of lesion volume exceeding a given z-score threshold and EDSS at baseline (A) and correlation between gradient of volume exceeding a given z-score threshold in NAWM at baseline (B) and EDSS assessed 2 years after baseline in patients with early multiple sclerosis (* indicates statistically significant values).

A								
	Correlation coefficient for the z-score thresholds				p-values			
Gradient between bands	0	1	2	3	0	1	2	3
1–2	0.292	0.279	0.282	0.290	0.047*	0.058	0.055	0.048*
1–5	0.392	0.368	0.374	0.377	0.006*	0.011*	0.010*	0.009*
1–10	0.402	0.396	0.377	0.386	0.005*	0.006*	0.009*	0.007*
1–20	0.389	0.379	0.378	0.375	0.007*	0.009*	0.009*	0.009*
1–30	0.375	0.360	0.365	0.371	0.009*	0.013*	0.012*	0.010*

B								
	Correlation coefficient for the z-score thresholds				p-values			
Gradient between bands	0	1	2	3	0	1	2	3
1–2	0.219	0.157	0.218	0.198	0.138	0.292	0.142	0.181
1–5	0.216	0.211	0.251	0.237	0.145	0.155	0.089	0.109
1–10	0.143	0.298	0.287	0.253	0.338	0.042*	0.050*	0.087
1–20	0.036	0.336	0.355	0.278	0.808	0.021*	0.014*	0.058
1–30	0.032	0.347	0.355	0.282	0.833	0.017*	0.014*	0.055

EDSS = expanded disability status scale, NAWM = normal appearing white matter.

### 3.3 Comparison of gradients of normalized NAWM voxels with z-score > 2 between all patients stratified according to physical disability (EDSS)

When the gradients were analysed separately for groups of patients with different levels of disability, we observed higher z-score derived metrics in more disabled patients in NAWM volume with z-score > 2 (Supplementary Fig. 3). However, a significant difference was observed only between minimally disabled patients (EDSS 0–1.5) and patients with moderate to severe disability (EDSS > 4).

## 4 Discussion

By using T1 relaxometry, we found a periventricular gradient of tissue damage that correlates better with physical disability than conventional MRI metrics. In contrast to previous studies demonstrating this tissue pathology gradient (Liu et al., 2015), we did not perform a group-wise comparison of absolute T_1_ relaxation times values, but rather used a single-subject z-score-based approach. The z-scores were derived from a study-specific atlas of healthy controls that takes age and sex into account (Piredda et al., 2020). This single patient approach has several advantages. Compared to direct comparisons based on absolute T_1_ values, the comparison between z-scores is not biased by differences in demographics between the subgroups – which is highly relevant due to changes of brain tissue T_1_ relaxation times over a lifetime. This approach ensures a high sensitivity to T_1_ signal pathology on a single-subject basis, enabling the detection of subtle alterations in tissue usually visualized as normal-appearing brain tissue by conventional MRI (Piredda et al., 2020).

### 4.1 Confirmation of a periventricular gradient of tissue damage

The degree of tissue damage reflected both by highest absolute z-score value as well and highest number of voxels with (z-score) > 2 was most pronounced in the tissue adjacent to lateral ventricles (in band 5; 5 mm from ventricles), and also in more disabled patients with progressive multiple sclerosis; it then decreased gradually with the distance from the ventricles. Our results are in line with previously published data highlighting the clinical relevance of the periventricular gradient of microstructural brain damage in the NAWM (Liu et al., 2015) (Pardini et al., 2016), even though the underlying mechanism has yet to be elucidated. There are several hypothetical explanations for the mechanism of periventricular tissue damage.

Not surprisingly, we observed a gradient of lesion probability with respect to distance from the ventricles. As the central vein sign is the hallmark of multiple sclerosis white matter lesions (Tallantyre et al., 2009), the density of veins is highest in the periventricular tissue (Narayanan et al., 1997), and the perivascular spaces of veins are continuous with subarachnoid space, a CSF-mediated damage might “spread” not only from ventricular ependyma but also from the perivascular spaces.

As it has been shown that this gradient is less pronounced after treatment with alemtuzumab (J. William L. Brown et al., 2020), it might be associated with partially reversible inflammatory activity. A recent PET study demonstrated one of the putative pathologic substrates for this gradient, consisting of a periventricular gradient of innate immune cell activation, reflected by [^18^F]-DPA714 binding that correlated with the degree of MTR abnormalities (Poirion et al., 2021). Similarly to our study, the gradient was observed not only in NAWM, but also in T2 lesions (Poirion et al., 2021). The authors speculate that the compartmentalized inflammation in periventricular lesions might be a source of proinflammatory mediators that diffuse into NAWM and activate innate immune cells, driving neurodegeneration and ultimately disability progression. The association of tissue damage and distance from CSF spaces has also been observed in the spinal cord of patients with early multiple sclerosis. The decrease of MTR in multiple sclerosis patients compared to healthy controls was not homogenously distributed. Again, the maximal changes were observed in the proximity of inner and outer CSF spaces (Combès et al., 2019), suggesting that a CSF-mediated tissue damage process takes place in the spinal cord as well.

### 4.2 The correlation between z-scores derived metrics and EDSS in early and progressive multiple sclerosis

In early multiple sclerosis, both the gradient of tissue damage in NAWM and the z-score derived metrics in WM lesions correlated better with physical disability assessed 2 years after baseline than conventional metrics, i.e., total lesion count and volume. The strength of correlation was similar to previous work correlating periventricular MTR gradient and EDSS(Brown et al., 2020a). We did not find a statistically significant association between these z-score derived metrics in NAWM and lesions and physical disability assessed at baseline. However, this is not surprising, due to the overall low EDSS scores that might be driven by a single first demyelinating attack in some patients. Also, it has been shown that in the early stages, MRI-derived metrics better predict the future clinical status than the current status (Bakshi et al., 2008). Firstly, this suggests that periventricular WM damage is at least partially independent of WM lesions in the same region (Brown et al., 2017). Secondly, the metrics may also yield new information refining the pathology within the lesions and thus correlate better with clinical involvement than lesions identified by the conventional sequence (FLAIR).

We observed higher correlations between gradient-derived metrics and EDSS in patients with progressive multiple sclerosis. In this cohort, with its more advanced disease and longer disease duration, physical disability was better reflected by the gradient of tissue damage in NAWM, and also by z-score derived metrics in WM lesions, than by conventional MRI-derived metrics. In the progressive course the blood–brain barrier has decreased permeability and so the inflammation is compartmentalized behind the blood–brain barrier, with less active lesions visible on conventional MRI. Because of this, it has been suggested that for progressive course monitoring it is better to use non-conventional techniques (Petracca et al., 2018), a suggestion supported by this study. Similar to our results, MTR in progressive multiple sclerosis patients showed more prominent heterogeneous reductions in both lesions and NAWM (Correale et al., 2017). A post-mortem study showed that the pathological substrates of quantitative MRI abnormalities in NAWM are dependent on distance from focal WM lesions. Close to visible lesions, axonal degeneration and activation of the microglia were associated with quantitative MRI parameters, whereas in the NAWM distant from lesions, the underlying pathology might be microglial activation associated with proximity to cortical lesions (Moll et al., 2011).

The correlation of lesional z-score metrics and EDSS is also in line with previous work which showed that the volume of lesions with very long T_1_ relaxation times (black holes) correlates better with composite clinical functional scores than total lesion volume. The decrease in T_1_-relaxation times inside black holes at follow-up was associated with clinical improvement and response to therapy (Thaler et al., 2015). Another recent concept that supports the hypothesis of CSF-mediated damage is the presence of so called “atrophied lesions”, areas of lesion tissue at baseline that change to ventricular CSF at follow-up (Dwyer et al., 2018). On the other hand, the presence of atrophied lesions might complicate the assessment of periventricular areas, i.e., the definition of the “first band”. Related to this, it is important to note that new and enlarging WM lesions close to the ventricular system have more impact on atrophy (lateral ventricle enlargement) than more distant lesions, further underlining the importance of understanding the mechanisms of periventricular tissue damage (Sinnecker et al., 2020).

Hypothetically, T_1_ times might reflect different processes in different lesion types and different disease stages. We have not explored the differences in T_1_ times with respect to lesion localization and have not differentiated between T_1_ hypo- and isointense lesions. Besides understanding the lesion heterogeneity in MS, T_1_-relaxation time gradient and gradients in surrounding NAWM could serve for differentiating other conditions with periventricular WM lesions, including ischaemic small-vessel disease (Wardlaw et al., 2013) and neuromyelitis optica spectrum disorder (Cacciaguerra et al., 2021) (Jurynczyk et al., 2017).

### 4.3 Limitations and future research directions

This study has a few limitations in relation to the generalizability of results. The somewhat polar patient distribution of either very early or progressive multiple sclerosis is not representative for a general multiple sclerosis population. Furthermore, in the progressive cohort we analysed SPMS and PPMS patients together. However, abnormal periventricular and cortical MTR gradients did not differ between PPMS and SPMS, suggesting comparable pathological processes (Brown et al., 2020a, Brown et al., 2020b).

Another limitation of our study is that the presence of possible atrophy in WM and deep GM concomitant to ventricle enlargement was not evaluated during the spatial registration of the patients’ brain anatomies. To minimize any possible bias due to difference in brain anatomy among patients, we non-linearly registered the patients’ MP2RAGE “uniform” contrasts to a study-specific template built from the healthy cohort. In doing so, the segmented bands around the ventricles correspond to the same anatomy among all patients. Future work should, however, investigate whether the potential presence of atrophy in the patient’s dataset may introduce bias in spatial registration. The investigation of changes in cortical atrophy in relation to T1-derived metrics and EDSS will also be addressed in the future.

We have also excluded infratentorial regions and the spinal cord, as it is difficult to measure white matter gradients there. Involvement of these regions seem to be associated with short- and long-term disease progression and disability in early multiple sclerosis (Giorgio et al., 2013) (Dekker et al., 2020) (Tintore et al., 2010), and isolated focal infratentorial lesions impact not only the integrity at the lesion site itself, but also along the entire affected fibre tract (Droby et al., 2015). Impact of these distant lesions on periventricular NAWM integrity should be considered in future studies. Finally, the cross-sectional design of our study does not allow us to investigate the periventricular gradient longitudinally, and especially how it may be affected by therapy. In untreated patients, periventricular gradient of MTR worsened over time. However, alemtuzumab has been shown to improve periventricular abnormalities (Brown et al., 2020a, Brown et al., 2020b). In the light of the CSF-mediated damage, it is tempting to speculate that this could drive the development of intrathecal therapies. Unfortunately, after intrathecal rituximab administration, the depletion of B-cells was lower than expected (Komori et al., 2016), and in progressive multiple sclerosis it failed to halt the progress of the disease and was associated with a risk of CNS infection (Bergman et al., 2021).

## 5 Conclusions

We have shown that a gradient in T_1_-derived tissue abnormality measures in both lesions and NAWM occurs in multiple sclerosis patients from the earliest stages of the disease, with the highest degree of pathology in the immediate periventricular region. As opposed to previous work on the “surface-in” gradient, we used z-score derived metrics that could allow use at the individual level. Gradients were already detectable at disease onset before treatment initiation, and thus have a potential to help in differential diagnosis. Metrics derived from the z-score gradient correlated with clinical disability in the progressive cohort and, in the early stages of disease, the baseline gradient parameters allowed for prediction of future disability. The relationship between z-score metrics and clinical disability is stronger when compared to lesion count and volume from conventional imaging. Therefore, in future the periventricular gradient in NAWM might qualify as a promising biomarker of multiple sclerosis from the early stages, and for monitoring in the progressive course.

## CRediT authorship contribution statement

**Manuela Vaneckova:** Conceptualization, Methodology, Formal analysis, Project administration, Writing – original draft. **Gian Franco Piredda:** Conceptualization, Software, Methodology, Formal analysis, Writing – original draft. **Michaela Andelova:** Methodology, Formal analysis, Writing – review & editing. **Jan Krasensky:** Writing – review & editing. **Tomas Uher:** . **Barbora Srpova:** Writing – review & editing. **Eva Kubala Havrdova:** Supervision, Writing – review & editing. **Karolina Vodehnalova:** Writing – review & editing. **Dana Horakova:** Writing – review & editing. **Tom Hilbert:** Project administration, Methodology, Writing – review & editing. **Bénédicte Maréchal:** Software, Methodology, Formal analysis, Writing – review & editing. **Mário João Fartaria:** Software, Formal analysis. **Veronica Ravano:** Methodology, Formal analysis, Writing – review & editing. **Tobias Kober:** Methodology, Conceptualization, Supervision, Formal analysis, Writing – review & editing.

## Declaration of Competing Interest

M. Vaneckova received speaker honoraria, consultant fees, and travel expenses from Biogen Idec, Novartis, Roche, Genzyme, and Teva, as well as support for research activities from Biogen Idec. G.F. Piredda, T. Hilbert, B. Marechal and T. Kober are employees of Siemens Healthcare AG, Switzerland. M. Andelova received financial support for conference travel from Novartis, Genzyme, Merck Serono, Biogen Idec, and Roche. J. Krasensky received financial support for research activities from Biogen Idec. T. Uher received financial support for conference travel and honoraria from Biogen Idec, Novartis, Roche, Genzyme, and Merck Serono, as well as support for research activities from Biogen Idec and Sanofi.B. Srpova received financial support for conference travel from Novartis, Genzyme, Merck Serono, Biogen Idec, and Roche. E. Kubala Havrdova received speaker honoraria and consultant fees from Biogen Idec, Merck Serono, Novartis, Genzyme, Teva, Actelion, and Receptos, as well as support for research activities from Biogen Idec and Merck Serono. K. Vodehnalova received compensation for travel, conference fees, and consulting fees from Merck, Sanofi Genzyme, Biogen Idec, and Novartis. D. Horakova received compensation for travel, speaker honoraria and consultant fees from Biogen Idec, Novartis, Merck Serono, Bayer Shering, and Teva, as well as support for research activities from Biogen Idec.
