# Supplementary material for: Periventricular gradient of T1 tissue alterations in multiple sclerosis
Source: Neuroimage Clin. 2022 Apr 16;34:103009. doi: 10.1016/j.nicl.2022.103009 (PMC9112026; doi:10.1016/j.nicl.2022.103009)
Supplement: Supplementary data 1 [file mmc1.docx]

**Supplementary material**


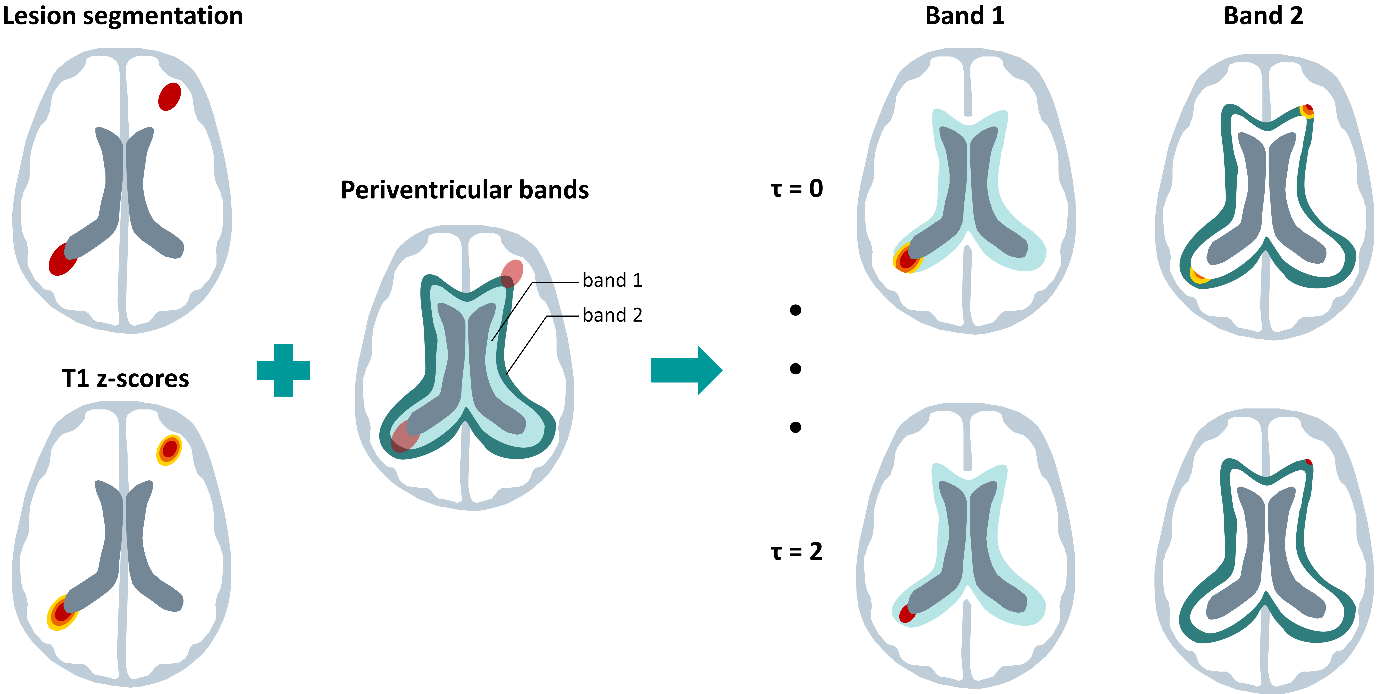


Supplementary Fig. 1. Lesion segmentation masks, T1 z-scores and periventricular bands are combined to extract for each band the volume of lesions exceeding a given z-score threshold (τ). The average z-score value is also extracted from these voxels. Two example bands are shown here. The same metrics are also extracted from the NAWM in each periventricular band.


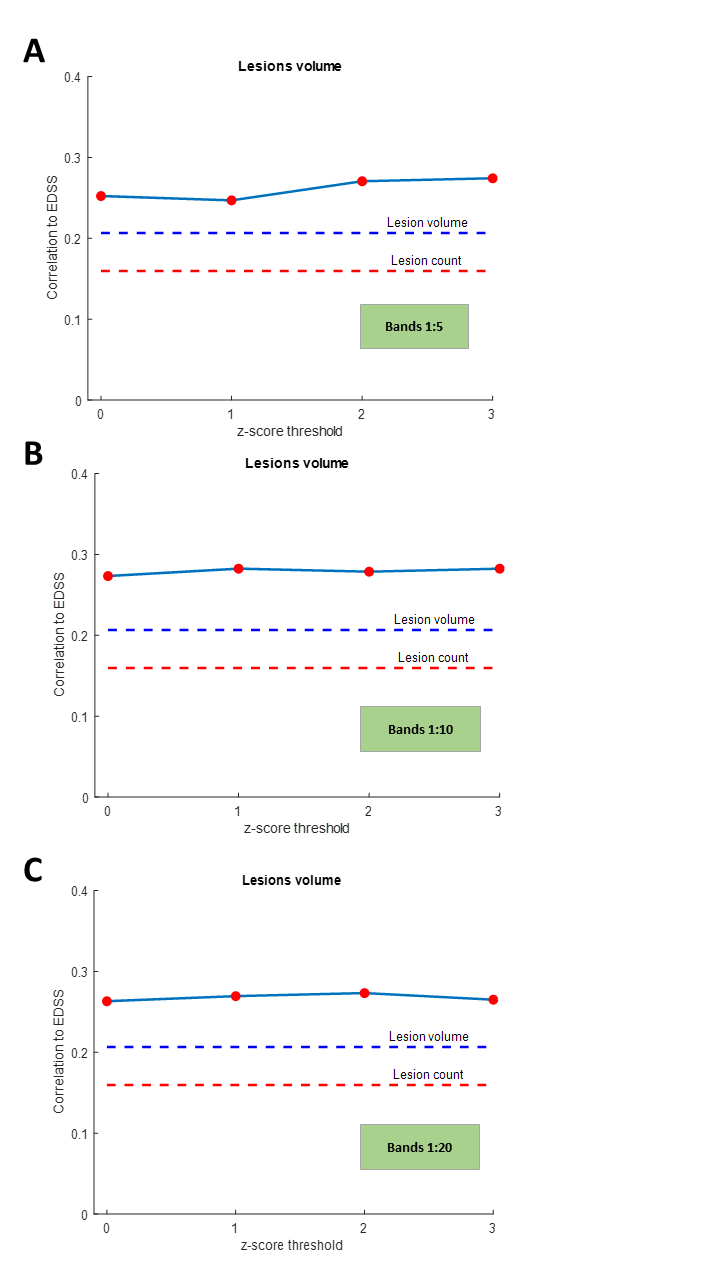


Supplementary Fig. 2. Correlation between gradient of lesion volume exceeding a given z-score threshold and EDSS (change between bands for z-score thresholds of 0, 1, 2 and 3) in patients with early multiple sclerosis: (A) band 1:5; (B) band 1:10; (C) band 1:20. (* indicates statistically significant values).

| Parameter | 3D MPRAGE | 3D FLAIR | 3D MP2RAGE | |
| --- | --- | --- | --- | --- |
|  |  |  | HC & early MS | Progressive MS |
| Resolution | 1.0x1.0x1.0 mm^3^ | 1.0x1.0x1.0 mm^3^ | 1.0x1.0x1.2 mm^3^ | 1.0x1.0x1.0 mm^3^ |
| Field of View | 256x256x176 mm^3^ | 256x256x176 mm^3^ | 256x256x212 mm^3^ | 256x256x224 mm^3^ |
| TI_1_/TI_2_ | 900 ms / - | 1800 ms / - | 700 ms / 2500 ms | |
| TE | - | 397 ms | - | |
| Flip angles | 9° | - | 4° / 5° | |
| TR | 2.3 s | 5 s | 5 s | |
| Undersampling | GRAPPA x2 | GRAPPA x3 | GRAPPA x3 | CS x4 |
| Bandwidth | 240 Hz/Px | 781 Hz/Px | 240 Hz/Px | |
| TA | 5:30 min | 3:17 min | 8:22 min | 4:35 min |

**Supplementary Table 1:** Details of the MRI protocols.

MPRAGE = magnetization prepared rapid acquisition gradient echoes; FLAIR = fluid attenuated inversion recovery; MPRAGE = magnetization prepared rapid acquisition gradient echoes; MP2RAGE= magnetization-prepared 2 rapid acquisition gradient echoes; HC = healthy controls; early MS = early multiple sclerosis; progressive MS = progressive multiple sclerosis; CS = Compressed sensing; GRAPPA = Generalized autocalibrating partially parallel acquisitions


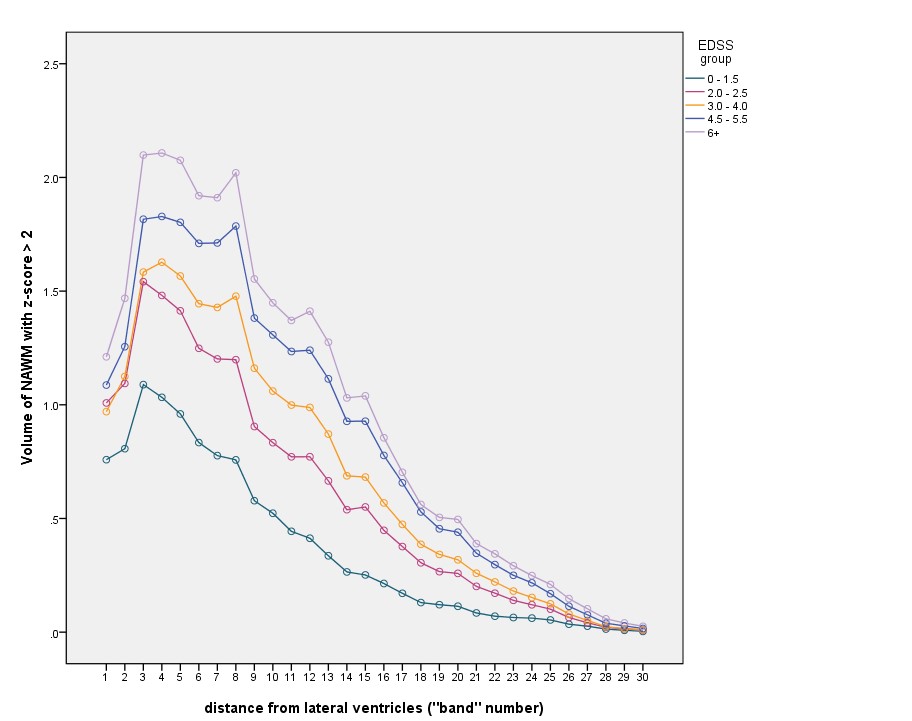


Supplementary Fig. 3. Comparison of gradients of normalized NAWM voxels with z-score > 2 between all patients, stratified according to physical disability (EDSS).
